# A Rare Presentation of Systolic Anterior Motion Occurring Eight Years After Mitral Valve Replacement

**DOI:** 10.7759/cureus.23114

**Published:** 2022-03-13

**Authors:** Olushola O Ogunleye, Hussain Dalal, Khalid Mahmood, Siyamek Neragi-Miandoab, Aarti Campo

**Affiliations:** 1 Department of Internal Medicine, Vassar Brothers Medical Center, Poughkeepsie, USA; 2 Department of Surgery, State University of New York (SUNY) Downstate Medical Center, Brooklyn, USA

**Keywords:** aortic regurgitation, mitral regurgitation, echocardiography, valve replacement, mitral valve, systolic anterior motion

## Abstract

Systolic anterior motion (SAM) is the dynamic displacement of mitral valve leaflets anteriorly toward the left ventricular outflow tract (LVOT) during systole. SAM-like physiology has been reported to occur shortly after mitral valve replacement (MVR) surgery; occurrence beyond two years after surgery is very rare. A 55-year-old woman who had bioprosthetic MVR eight years earlier for non-rheumatic mitral stenosis presented to the emergency room with progressive dyspnea and sudden-onset chest pressure. Physical examination noted a grade 3/6 systolic murmur at the cardiac apex, a soft diastolic murmur at the left sternal border, and diffuse expiratory wheezing. B-type natriuretic peptide (BNP) was elevated (286 pg/mL). Transthoracic echocardiography (TTE) showed mitral regurgitation and severe aortic insufficiency; the mitral prosthesis was protruding into the LVOT, causing LVOT obstruction with a peak gradient of 16.3 mmHg and peak velocity of 2.0 m/s. Transesophageal echocardiography (TEE) confirmed severe bioprosthetic MV dysfunction, severe aortic regurgitation, and SAM-like physiology. Left cardiac catheterization showed normal coronaries. She underwent repeat MVR and aortic valve replacement. She was started on daily aspirin and warfarin post-operatively, then discharged home on post-operative day 10. During post-operative office visits, she reported no complications. SAM-like physiology was absent in a two-month follow-up TTE, with reduced LVOT peak gradient of 6.5 mmHg and peak velocity of 1.3 m/s. Dynamic SAM-induced LVOT obstruction could be asymptomatic or result in severe heart failure with 20% risk of sudden cardiac death. SAM may occur within days following MVR or may have a delayed presentation. Medical management, including beta-blockade, is the cornerstone of initial management, while structural damage to the prosthetic valve mandates repeating mitral valve replacement surgery. This case highlights the importance of long-term follow-up of patients after MVR to assess for SAM, which could occur with or without degenerative changes of the prosthetic valve.

## Introduction

Systolic anterior motion (SAM) is the dynamic displacement of mitral valve (MV) leaflets anteriorly toward the left ventricular outflow tract (LVOT) during systole [1-3]. SAM was initially considered only in hypertrophic cardiomyopathy, but it is now known to be induced by multiple conditions that alter the dynamic left ventricular anatomy/physiology [1,2]. SAM has also been reported after mitral valve surgeries [1,4,5]. Furthermore, SAM has been reported in healthy adults loaded with dobutamine during stress echocardiography, or with the use of inotropes in cases of hypovolemic shock or during general anesthesia [1,2,5,6]. In relation to mitral valve surgeries, most cases of SAM occur in the early post-operative period. We present a case of SAM occurring eight years after mitral valve replacement (MVR), requiring repeat MVR.

## Case presentation

The patient is a 55-year-old Caucasian woman with atrial fibrillation (on apixaban and diltiazem), active smoking, chronic obstructive pulmonary disease, hyperlipidemia, coronary artery disease, and hypertension. Eight years earlier, she underwent coronary artery bypass grafting and bioprosthetic MVR with Magna Ease tissue (Irvine, CA: Edwards Lifesciences) at a different hospital for non-rheumatic mitral stenosis (additional surgical details unavailable). She presented to the emergency department at our hospital with a one-week history of intermittent mid-sternal chest discomfort and dyspnea on exertion, then sudden onset of palpitations, diaphoresis, and light-headedness. At presentation, she was tachycardic (147 beats per minute {bpm}) and normotensive (129/87 mmHg). Electrocardiography showed 2:1 rapid atrial flutter. Troponin and electrolyte levels were within normal limits, and chest radiograph did not show any features suggestive of volume overload. Following failed attempts at rate control with IV diltiazem and IV metoprolol, she underwent direct current cardioversion. Post-cardioversion, she developed transient hypotension which normalized after a bolus of 1 L of normal saline. The patient was planned for hospital admission, but she demanded to be discharged home against medical advice.

**Figure 1:**
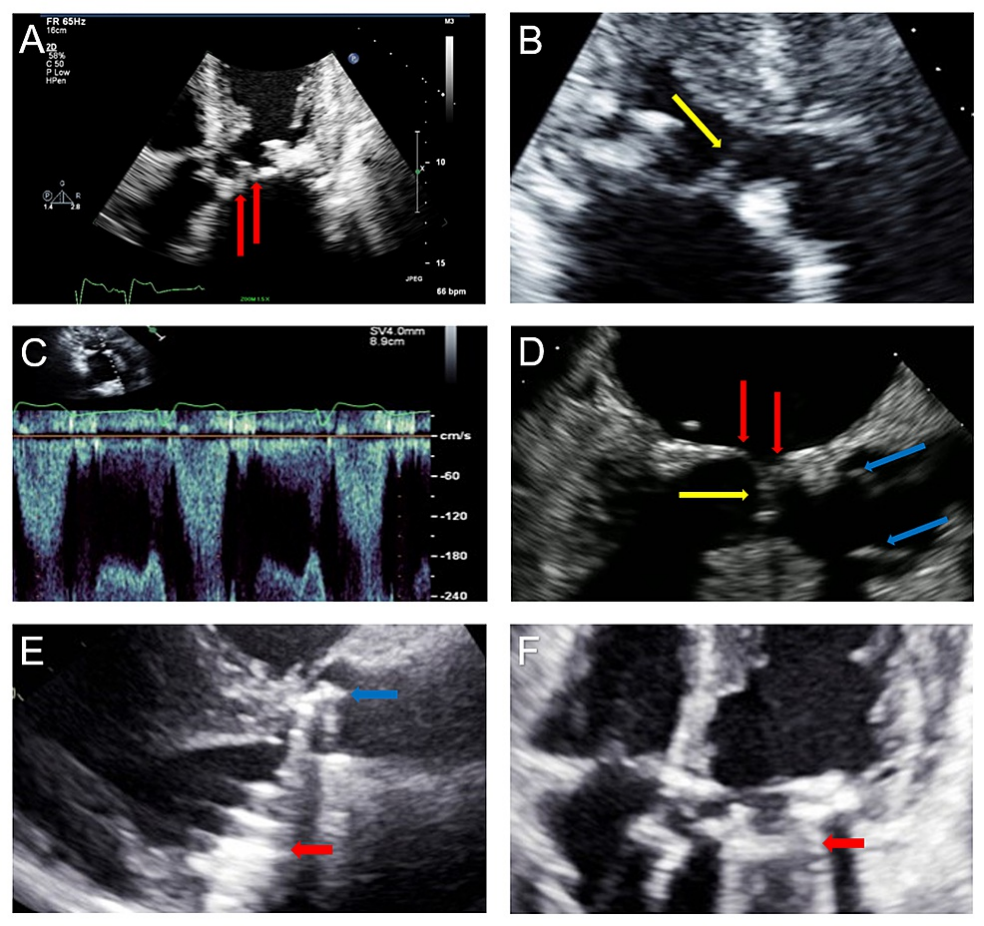
Multimodality echocardiography. (A) Apical five-chamber view of mitral valve bioprosthetic leaflets (red arrows) at start of diastole. (B) Parasternal long-axis view of the mitral valve leaflets in LVOT (yellow arrow) during systole. (C) Pulse wave spectral Doppler at the LVOT showing elevated subvalvular velocity. (D) Pre-operative transesophageal echo. Transducer angle 150° shows the mitral valve (red arrows) during systole, with anterior mitral valve leaflet protruding into the LVOT (yellow arrow), while the aortic valve is open (blue arrows). (E) Two-month post-operative echo (parasternal long-axis view). Red arrow represents the mechanical mitral valve; blue arrow represents the mechanical aortic valve. (F) Two-month post-operative echo (four-chamber view). Red arrow represents the mechanical mitral valve. LVOT: left ventricular outflow tract

The following day, the patient returned to the emergency department with worsening shortness of breath, including dyspnea at rest, and sudden-onset of pressure-like chest pain radiating to her neck. Physical examination was notable for blood pressure of 126/84 mmHg, heart rate of 75 bpm, 3/6 holosystolic murmur at the apex, a soft diastolic murmur at the left sternal border, and diffuse expiratory wheezing. Laboratory evaluation was remarkable for elevated B-type natriuretic peptide (BNP) (286 pg/mL) and negative serial troponin-I (<0.03 ng/mL). Electrocardiography showed normal sinus rhythm with no ischemic changes. The patient was admitted to the cardiology unit. Transthoracic echocardiography showed an ejection fraction of 55-60% with severe mitral regurgitation, severe pulmonary hypertension, and severe aortic insufficiency with the mitral prosthesis struts and leaflets protruding into the LVOT, thereby causing SAM-like physiology and LVOT obstruction with a peak gradient of 16.3 mmHg, and a peak velocity of 2.0 m/s (Figure 1, panels A-C). On day two, transesophageal echocardiography confirmed severe bioprosthetic MV dysfunction, a tri-leaflet aortic valve (AV) with severe regurgitation, and SAM-like physiology (Figure 1, panel D). Left cardiac catheterization showed no angiographically significant disease in the patient’s native coronary arteries, but she had an occluded saphenous vein graft bypass to the right coronary artery and a nearly obliterated left internal mammary artery graft to the left anterior descending artery.

The consensus following a multidisciplinary heart team meeting and discussion with the patient was that the patient should undergo surgical intervention with repeat MVR and AV replacement using mechanical valves. Her pre-operative workup (including carotid ultrasound and CT of the chest, abdomen, and pelvis) did not show any findings to preclude surgical intervention. Hence, on day five, the patient underwent repeat MVR (with On-X 25 standard) and aortic valve replacement (with On-X 19). Intra-operative findings included severe mitral annular calcification, an oversized bioprosthetic MV protruding into the LVOT, and a large septal bulge. The patient tolerated the surgery well and was started on daily aspirin and warfarin. She was discharged home on post-operative day 10 in stable condition. During her post-operative office visits, she reported improved exercise capacity and no complications. SAM-like physiology was absent in her two-month post-operative echocardiography, with reduced LVOT peak gradient of 6.5 mmHg, peak velocity of 1.3 m/s, and normal pulmonary artery pressure (Figure 1, panels E and F).

## Discussion

The mitral valve consists of anterior and posterior leaflets, the annulus, the subvalvular apparatus of the papillary muscles, and chordae tendineae. Normal functioning of the MV depends on the integrity of these structures as well as on the global left ventricular structure and contractility. The pathophysiology of SAM is multifactorial, with multiple studies attributing roles to the mitral leaflets, the mitral annulus, the subvalvular apparatus, and ventricular morphological changes [1,2]. The first known report of SAM that occurred as a complication of MV-related surgery was documented by Termini et al. in 1977 [4]. Since then, multiple reports and studies have documented its association with mitral valve surgery, but the pathophysiology is still not fully understood [2]. However, two major risk factors for SAM following MV reconstruction have been proposed: excessive valve leaflet tissue as in Barlow’s disease and implantation of a prosthesis whose ring or band is too small [7-9].

Dynamic SAM-induced LVOT obstruction occurs in about 4-11% of patients after MV repair or replacement [2,10,11]. Patient presentation ranges from clinically quiescent disease to severe hemodynamic compromise [2]. SAM can result in severe heart failure and has a 20% risk of sudden cardiac death [1]. Presence of a peak resting LVOT gradient >30 mmHg has been identified as a risk factor for progression to heart failure or sudden cardiac death [10]. Our patient’s peak LVOT gradient was below this threshold, and she did not have signs consistent with heart failure. SAM may occur within days following MVR or may have a delayed presentation, as late as one to two years. Presentation of SAM beyond two years post-MVR is very rare. To our knowledge, from review of the current literature, there are only two reported cases of SAM occurring up to eight years after MV repair or replacement [8,12].

Bioprosthetic MVs have a durability of 10-15 years before deteriorating and requiring replacement [13]. Implantation of prosthetic MVs with rings sizes ≥36 mm has been advocated as a measure to prevent mitral stenosis after MVR [7,9]. However, as seen in our patient, the use of oversized valve can be associated with SAM physiology and early deterioration of the valve [12]. The treatment approach is guided by the severity of symptoms [1,2,5,11]. In most cases of SAM, symptoms improve with conservative measures including beta-blockade, hydration, and vasoconstriction. Indications for surgical intervention include severe or worsening symptoms or structural damage to the MV [1,2,5,11]. Besides SAM physiology, our patient had additional indications for repeat surgery, including severe prosthetic mitral regurgitation and severe aortic insufficiency.

## Conclusions

Considering that the LVOT gradient was not very high at presentation, our patient’s symptoms were likely multifactorial in etiology - involving a combined effect of her SAM physiology, her severe mitral regurgitation, and her severe aortic regurgitation. This case highlights the importance of long-term follow-up of patients after MVR to assess for SAM, which could occur with or without degenerative changes of the prosthetic valve.
